# A Rare Case Report of Granular Cell Tumour of the Abdominal Wall and a Review of the Literature

**DOI:** 10.7759/cureus.54399

**Published:** 2024-02-18

**Authors:** Simone H Mangan, Jessica Y Ng, Philip Townend

**Affiliations:** 1 Department of General Surgery, Gold Coast University Hospital, Gold Coast, AUS; 2 School of Medicine, Griffith University, Gold Coast, AUS; 3 Department of Surgery, Gold Coast University Hospital, Gold Coast, AUS

**Keywords:** rare soft tissue tumours, en bloc surgical resection, case report, abdominal wall, granular cell tumors

## Abstract

Granular cell tumours (GCTs) are rare soft tissue tumours of neural origin. They have been reported in multiple anatomical sites. However, only 14 cases worldwide have been reported arising from the abdominal wall. While they can clinically manifest in a variety of ways, often they present as a small, slow-growing nodule with benign features. They can, however, be malignant, and in rare cases, they have been reported to metastasise. Here, we present a case of a rare abdominal wall GCT, which was managed with local excision. The purpose of this paper is to report the patient’s clinical history, presentation, and surgical management, as well as to review the current literature to highlight the existence of this rare entity and the possibility that this may occur and should be considered a differential diagnosis in clinical practice.

## Introduction

Granular cell tumours (GCTs) were first described in 1854 by Weber and Virchow [1]. Rekhi et al. identified them as a unique category of mesenchymal soft tissue tumours that were initially thought to be of skeletal origin. However, they were later confirmed to be of neural origin, specifically from Schwann cells [2]. These tumours have been found in various anatomical locations, including the peripheral soft tissues of the trunk, head, and neck and, internally, with the commonest site being the oesophagus [3-5]. They constitute less than 50% of all soft tissue tumours and are even rarer in the abdominal wall.

According to the Fanburg criteria, GCTs can be classified as benign, atypical, or malignant based on a set of six histological parameters: necrosis, spindling, vesicular nuclei with large nucleoli, increased mitotic activity, high nuclear to cytoplasmic ratio, and the presence of pleomorphism [3]. GCTs can affect both genders but have a higher incidence in females aged 30-50 years and are more prevalent among African-Americans compared to Caucasians [1,4]. They are more commonly found in middle-aged adults but can occur across various age groups [4-7].

We report here the first published case in Australia of a patient with an abdominal wall GCT, which was found incidentally as an abdominal wall lump. It is important to report these rare cases to add to the literature so it is known and considered in clinical practice. The diagnosis was made with a combination of imaging and en bloc resection to allow a histopathological assessment.

## Case presentation

**Figure 1:**
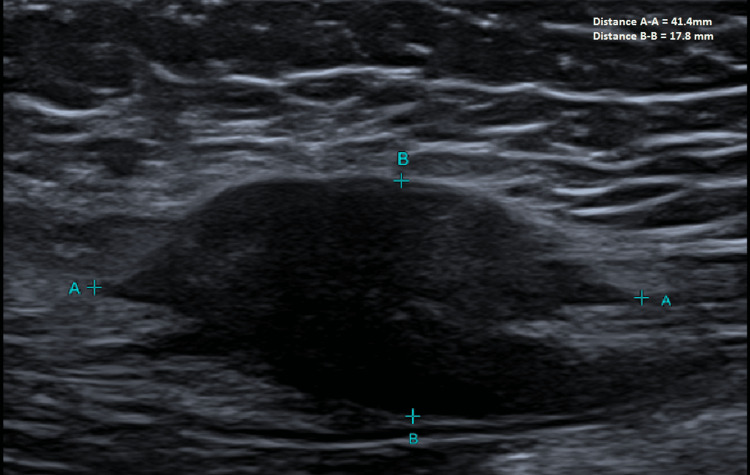
Ultrasound scan of the left abdominal wall demonstrating a hypoechoic mass within the soft tissue of the abdominal wall.

**Figure 2:**
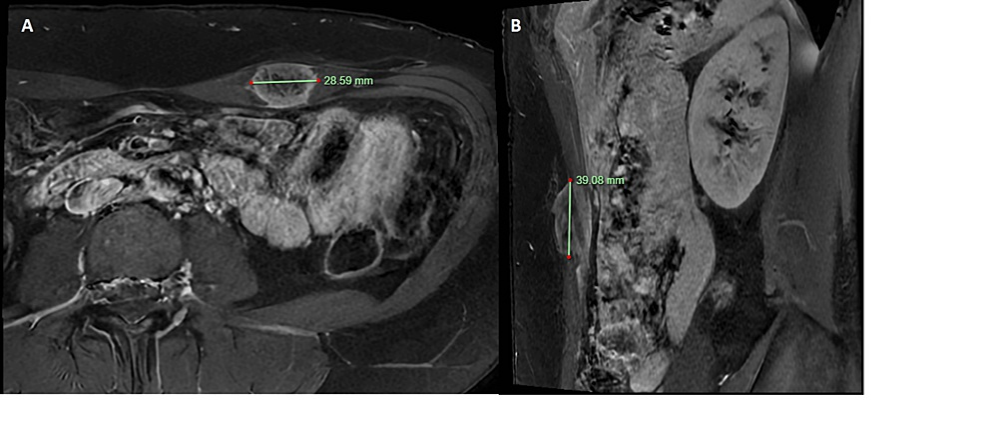
Magnetic resonance image of the patient's abdominal wall showing T1 phase post-contrast in (A) axial and (B) sagittal views. The tumour can be seen as a hypointense mass within the left rectus abdominis muscle with contrast rim enhancement.

A 43-year-old Caucasian female presented to her general practitioner for a routine checkup. A 3 x 3 cm abdominal wall lump was found incidentally on routine clinical examination. She was completely asymptomatic and had no previous medical history. She was otherwise well and worked as a swimming instructor. Ultrasound (US) imaging revealed a 4.1 x 0.8 x 0.88 cm heterogeneous hypoechoic mass that had irregular spiculated posterior margins. It was reported to be confined to the subcutaneous tissues of the left anterior abdominal wall. Infiltration into the adjacent soft tissues was also reported (Figure 1). It was further characterised by magnetic resonance imaging (MRI), which reported a 2.6 x 1.7 x 3.4 cm well-defined intramuscular nodule within the left rectus abdominis muscle without invasion into the underlying abdominal cavity or overlying subcutaneous tissue (Figure 2). A computed tomography (CT) chest did not show any metastatic lesions.

Clinical examination revealed an approximately 3 x 3 cm, firm, non-mobile lump in the left upper quadrant of the patient’s abdomen. There were no palpable locoregional lymph nodes. A core needle biopsy showed small clusters of large cells present with abundant granular cytoplasm and small round nuclei. There was no necrosis or increase in mitotic rate. The stains were positive for S100, SOX0, CD68, and cyclin D1. It was concluded that the biopsy result favoured a GCT.

**Figure 3:**
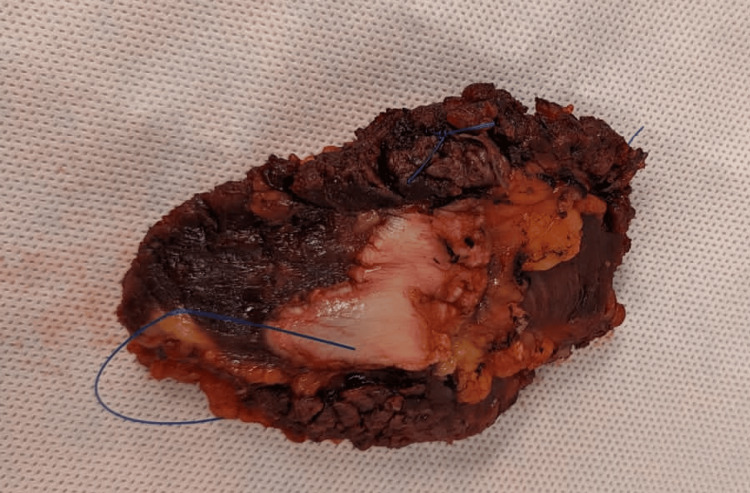
The granular cell tumour post en bloc excision. The specimen measured 8.3 x 5.3 x 4.3 cm to obtain adequate resection margins and included skin, fat, and rectus abdominus skeletal muscle.

Based on the aforementioned investigations, it was clinically decided that the patient required an open-wide local excision of the lesion, as well as a planned reconstruction of the abdominal wall. The lesion was excised with a cuff of muscular tissue, as well as the posterior compartment of the rectus sheath en bloc with wide margins to maximise complete resection (Figure 3). The posterior sheath was closed, and the anterior sheath was partially closed. The resulting 5 x 3 cm defect was bridged with a composite mesh (ParietexTM).

The patient had a routine post-operative course, without any acute complications. She was discharged home the following day. At her two-week follow-up review, the wound had fully healed, with no clinical concerns of a hernia or complications, confirming the successful excision of the GCT and, to date, an intact reconstruction of her abdominal wall. Histopathological and immunohistochemical staining confirmed the diagnosis of a 4 x 2 x 2.7 cm abdominal wall GCT without any malignant features, confirming a benign GCT, as per the Fanburg-Smith criteria (Table 1) [3]. The lesion was completely excised with clear margins.

**Table 1:** Fanburg-Smith diagnostic criteria for malignant granular cell tumours. # at 200x magnification

Histologic Feature	Number of Features Present		
	Benign	Atypical	Malignant
Necrosis	0	1-2	≥ 3
Spindling			
Vesicular nuclei with large nucleoli			
Increased mitotic activity >2 mitoses/10 high-power fields^#^			
High nuclear to cytoplasmic (N:C) ratio			
Pleomorphism			

## Discussion

Abdominal wall GCTs are extremely rare, with only 14 cases reported worldwide (Table 2). They often present as painless, subcutaneous nodules [3,6]. They can also present as small, slow-growing, solitary nodules that generally behave in a benign fashion but have a tendency to recur [1]. With the addition of the case we report here, the average age of patients with abdominal wall GCTs is 51.4 years and occurs far more commonly in females than males, with 86.7% (13/15) of cases being reported in females. The reason for this gender discrepancy is not known. However, it is consistent with the prevalence of all GCTs, not just those occurring in the abdominal wall, and is an area needing further investigation. Histologically, these tumours are characterized by large cells with abundant granular cytoplasm [3,6]. Immunohistochemical studies often reveal positivity for S100 protein, neuron-specific enolase (NSE), and the histiocytic marker CD68, confirming their neural origin [2-4,6]. Clinical features can vary widely, with some cases reporting a hard mass fixed to the deep muscle layer [2,3,7].

**Table 2:** Summary of reported abdominal wall granular cell tumours in the literature. cm = centimetres, M = male, F = female, B = benign, M = malignant

Age/Gender	Clinical Features and Symptoms	Tumour size (cm)	Management	Surgical Resection Margin	Pathology B/M	Recurrence Rates and Follow-up	Reference (Year)
58/F	Incidental finding	8 x 4	-	-	B	-	Gorelkin et al. (1978) [8]
30/F	-	9	Surgical excision	-	M	-	Vamsy et al. (1992)[9]
44/F	Non-tender, hard mass right lower abdominal wall, four months	3.6 × 2.5	Surgical excision	Completely excised	B	Not stated	An et al. (2007) [10]
70/F	Abdominal pain, diarrhoea, weight loss, palpable mass	4	En bloc resection of the abdominal wall tumour. Use of Permacol® mesh for closure.	Completely excised	B	Alive, well at five-month review	Chaudhry et al. (2008) [11]
49/F	Non-tender palpable mass left upper quadrant abdominal wall	2.1	En bloc wide local excision	Completely excised	B	Not stated	McGhan et al. (2012) [12]
29/F	Hard, fixed to the deep muscle layer, no connection to the skin.	1.2 x 0.9 x 1	En bloc excision performed	Not stated	B	No recurrences reported up to 3 years after surgery	Panunzi et al. (2012) [13]
45/F	Light pain in upper abdominal wall, six months	1.6 x 3	En bloc surgical	Completely excised	B	Not stated	Porta et al. (2015) [1]
50/F	Painless, palpable mass abdominal wall, > 12 months	0.5 x 1 x 1.5	Surgical excision	Not stated	B	Well after 1 week of review	Wang et al. (2015) [14]
66/M	Palpable abdominal wall mass for 30 years, recent increase in size.	4.5x3.4 x 3	Surgical excision	Not stated	M	Alive and well, no recurrence at 30 months	Yoon et al. (2016) [4]
60/F	Incidental finding	Not stated	Laparoscopic-endoscopic cooperative surgery for gastric submucosal tumour	Not stated	B	Alive and well at 12-month review	Saito et al. (2018) [7]
50/F	Enlarging peri-umbilical mass over 12 months	7 x 6	Surgical excision	Completely excised	M	Surveillance under oncology. CTs at 8mo, 2, 3, and 4 years showed unilateral lung and groin nodules. Commenced palliative chemotherapy. Lesions continued to increase over time. Alive, nodules stable at 11-year review	Alnashwan et al. (2019) [6]
37/M	Palpable abdominal wall lump	2.7 x 2 x 1.6	Surgical excision	Not stated	B	Not stated	Joshi et al. (2003) [15]
67/F	Abdominal pain	10	Surgical excision	-	M	-	Chelly et al. (2005) [16]
73/F	Painless palpable abdominal wall mass	2.4x 2.3x 1.3	Wide local excision under local anaesthetic	Not stated	B	Well at 8 months	Rehan et al. (2021) [17]

Diagnostic investigations typically include US and CT scans to assess the size and extent of the tumour [5,7]. MRI is considered the best radiological modality for characterising GCTs [1]. Of the 14 cases reported, those who underwent US also underwent a CT or MRI and were 49 years of age or younger [1,12,17]. One patient who had an incidental submucosal gastric lesion and an abdominal wall GCT underwent a CT, MRI, and Fluorine-18 fluorodeoxyglucose positron emission tomography (18F-FDG PET). The abdominal wall GCT, in this case, was avid with a maximum standardised uptake value (SUV max) of 1.92 [7]. The radiologic characteristics of malignant GCTs (MGCTs) are not well-understood, making it difficult to differentiate them from other soft tissue lesions, such as desmoid tumours [4]. Fine-needle aspiration cytology (FNAC) may be performed, although its utility is limited due to the granular nature of the tumour [2,6,18]. Histopathological examination remains the gold standard for diagnosis [2,3,6].

The size of abdominal wall GCTs appears to be highly variable, ranging from 1 cm to 10 cm [5,16], and the surrounding skin may be thickened or hyperpigmented [4]. In rare instances, they can metastasise, particularly if they are deep to the fascia or larger than 4 cm [1]. There has been a report of a GCT of the thigh metastasising to the abdominal wall; in this case, the primary tumour was 10.5 cm and had not been completely excised [19]. The metastatic lesions to the abdominal wall and lungs occurred within five months of the initial resection. This case highlights the need to consider GCTs in the differential, as most are benign; however, some may be malignant and aggressive. The literature on the staging of GCTs is sparse, and there is currently no standardised staging system specific to GCTs [6,20]. However, diagnosis and management often involve imaging studies and histopathological examination [2,3,18,20].

The primary treatment for abdominal wall GCTs is surgical excision, posing unique challenges due to their location [6,11]. Wide local excision with clear margins is recommended to minimize the risk of recurrence [2,6,11]. However, the structural integrity of the abdominal wall and reconstructive options must be considered [6,11]. In one report, surgical reconstruction of the abdominal wall was successfully performed using a biosynthetic porcine mesh after tumour excision [11]. Local surgical excision, if complete, is curative for benign GCTs, and wider local excision may be recommended if resection margins are involved [1,3].

## Conclusions

GCTs of the abdominal wall are extremely rare but should not be overlooked. Diagnostic procedures, such as CT scans and histopathological examination, are crucial for an accurate diagnosis. Surgical excision, with clear margins, remains the primary treatment goal. When they occur in the abdominal wall, they have the added complexity of the need for consideration of reconstruction of the abdominal wall following excision.

This case report presents an example of a primary GCT in the abdominal wall. With only 14 reported cases, it highlights the importance of being aware of this type of soft tissue tumour to report it to add to what has already been published and to highlight how to investigate and treat it once identified. Further research is required in identifying radiological and clinical features of GCTs that can differentiate benign from malignant tumours.
